# Immobilisation of α-amylase on activated amidrazone acrylic fabric: a new approach for the enhancement of enzyme stability and reusability

**DOI:** 10.1038/s41598-019-49206-w

**Published:** 2019-09-03

**Authors:** Ahmed R. Al-Najada, Yaaser Q. Almulaiky, Musab Aldhahri, Reda M. El-Shishtawy, Saleh A. Mohamed, Mohammed Baeshen, Ammar AL-Farga, Wesam H. Abdulaal, Sami A. Al-Harbi

**Affiliations:** 1 King Abdulaziz City for Science and Technology, P.O. Box 6086, 11442 Riyadh, Saudi Arabia; 2 Chemistry Department, Faculty of Sciences and Arts, University of Jeddah, Khulais, P.O. Box 355, Khulais, 21921 Saudi Arabia; 3 Chemistry Department, Faculty of Applied Science, Taiz University, Taiz, Yemen; 4 Department of Biochemistry, Faculty of Science, King Abdulaziz University, Jeddah, P. O. Box 80200, Jeddah, 21589 Saudi Arabia; 5 Center of Nanotechnology, King Abdulaziz University, Jeddah, 21589 Saudi Arabia; 6 Chemistry Department and Faculty of Science, King Abdulaziz University, Jeddah, P. O. Box 80200, Jeddah, 21589 Saudi Arabia; 7 Dyeing, Printing and Textile Auxiliaries Department, Textile Research Division, National Research Center, Dokki, 71516 Cairo Egypt; 8 Molecular Biology Department, National Research Centre, Giza, Cairo Egypt; 9 Department of Biology, Faculty of Science, University of Jeddah, P.O.Box 80203, Jeddah, 21589 Saudi Arabia; 10 Department of Biochemistry, Faculty of Science, University of Jeddah, P.O.Box 80203, Jeddah, 21589 Saudi Arabia; 11 Department of Chemistry, University College in Al-Jamoum, Umm Al-Qura University, Makkah, Saudi Arabia

**Keywords:** Immobilized enzymes, Biophysical methods

## Abstract

In this study, amidrazone acrylic fabric was applied as an immobilising support for α-amylase. The immobilised α-amylase was characterised by Fourier transform infrared spectroscopy and scanning electron microscopy. Furthermore, the optimum conditions for immobilisation efficiency, immobilisation time, reusability, kinetic parameters and pH, for the immobilisation process were examined. The study demonstrated that with 4% cyanuric chloride, and a pH of 7.0, the highest immobilization efficiency of 81% was obtained. Around 65% of the initial activity was maintained after storage at 4 °C for 8 weeks. The immobilised enzyme retained 53% of its original activity after being reused 15 times and exhibited improved stability compared with the free enzyme in relation to heavy metal ions, pH, temperature and inhibitors. The immobilised enzyme presented kinetic parameters of 2.6 mg starch and 0.65 µmol maltose/mL for *K*_m_ and *V*_max_ respectively, compared with 3.7 mg starch and 0.83 µmol maltose/ mL for the free enzyme. The improvements in the enzyme’s catalytic properties, stability and reusability obtained from immobilisation make amidrazone acrylic fabric support a good promising candidate for bio-industrial applications.

## Introduction

Enzymes have found a place in modern biotechnology as biocatalysts in many industries, including medicine, energy and food. They play important roles in green chemistry and developments therein. Given their multiple critical properties, including the use of mild reaction conditions, low toxicity, good selectivity and lack of secondary reactions, they are used more frquenty than other traditional chemical processes^1,2^. α-Amylase (1,4-D-glucan glucanohydrolase) catalyses the hydrolysis of the α-D-(1,4) glycosidic linkages of glycogen, starch, and other oligosaccharides, and is found in microorganisms, mammalian tissues and plants^3,4^. These enzymes play important roles in industry and are used many fields, including paper, textiles, food and baking, and others^5,6^. However, the industrial application of amylases is confined by their short lifetime sensitivity to environmental factors, poor operational storage stability and high production costs which leads to difficulties in their reuse and recovery^7,8^. Therefore, for the large-scale industerial use of amylases, their stability needs to be improved via immobilisation by different physiochemical treatments^9^. Immobilisation defined as attaching enzymes to a support so that a reduction or loss of mobility maybe achieved with catalytic activity retention, thereby allowing continuous use of the enzyme. This approach allows for improved stability of the biocatalyst, easier separation of substrates and products, and recovery of the enzyme so that it can be used again^6,10,11^.

Support materials can be categorised as either organic or inorganic, depending on their chemical composition. The best support materials are inert towards the supported enzymes, low cost, physically resistant to compression, resistant to microbial attack and hydrophilic with an appropriate surface area, alongside minimal restriction of substrate diffusion. Immobilising support plays a substantial role in the immobilised enzyme’s performance. If immobilisation is to be achieved through the use of a surface binding technique, the bonding strength is strongly dependent on the physical link or chemical type used^12^. Acrylic fabrics are commonly used as enzyme supports, such as Eupergit C, which has been successfully used in industry for covalent attachment to many enzymes^13^.

Hydrazine is a ligand that can be used to create complexes. It is an area of increasing research interest and has been studied by Audrieth and Ogg ^14^, Bottomley^15^, Schmidt^16^ and Heaton^17^. Hydrazine is also of great academic interest because of its versatility, which is attributed to the existence of two free electron pairs and four replaceable hydrogen atoms. Its difunctional nature allows for many uses, including dibasic ligand reactions, which gives polymeric amides that can be drawn into fabrics. It can also be used in polymeric materials because of its cross-linking properties. In this study, amidrazone acrylic fabric activated with different concentrations of cyanuric chloride is used as a support for α-amylase by cross-linking methods. Moreover, the immobilised and free enzymes are characterised and compared. This is the first investigation of the cross-linking immobilisation of α-amylase on amidrazone acrylic fabric.

## Materials and Methods

### α-Amylase

α-Amylase from *Bacillus subtilis*, and other materials were purchase from Sigma-Aldrich and then used as obtained.

### Acrylic fabrics

A 1/1 woven acrylic acquired from El-Mehalla Co. in Egypt was used in this work. The fabric was washed with ethanol and then dried at ambient temperature.

### α-Amylase assay

To determine the activity of the immobilised and free enzymes, we opted for a technique outlined by Miller^18^. A 1 cm^2^ piece of activated acrylic fabric was used for the routine assay of the activity of immobilised enzyme. Immobilised α-amylase was removed after 30 minutes’ incubation with 1% starch (1 mL) at 37 °C and 1 mL of dinitrosalicylic acid (DNS) was included to help develop colour. The tube containing this mixture was then incubated for 10 minutes in a boiling water bath before cooling then the absorbance was recorded at 560 nm.

### Preparation of support

Acrylic fabric of a certain weight was pretreated with 1% hydrazine hydrochloride in the presence of 2% ammonium acetate at a liquor ratio of 50:1 at 85 °C for 60 minutes with shaking. Then, the modified acrylic fabric was completely rinsed with water and air-dried. The pretreated fabric was then activated with cyanuric chloride at different levels (2–6% w/w) in an acetone/water mixture (50% v/v) at pH 4.0 and 0 °C for 2 hours. Subsequently, the fabric support was thoroughly rinsed with cold water and, acetone then dried in a ventilated refrigerator prior to α-amylase immobilisation.

### Immobilisation procedure

The treated fabric was used as a support for the immobilisation of α–amylase dissolved in 50 mM Tris–HCl buffer at pH 8.0, or 7.0, or 50 mM sodium acetate buffer at pH 4.0. The reaction was carried out at room temperature overnight. The fabric was then allowed to dry at room temperature. To confirm the progression of the immobilization, the relative activity (%) was determined by using the following equation:

$${\rm{Relative}}\,{\rm{enzyme}}\,{\rm{activity}} \% =\frac{{\rm{Activity}}\,{\rm{of}}\,{\rm{immobilized}}\,{\rm{enzyme}}}{{\rm{Initial}}\,{\rm{activity}}\,{\rm{of}}\,{\rm{enzyme}}}\times 100$$

### Morphological characterisation

The attenuated total reflectance Fourier transform infrared (ATR –FTIR) spectra of the immobilised enzyme were obtained using a PerkinElmer Spectrum 100 FTIR spectrometer. The morphology of the immobilised enzyme was examined with a scanning electron microscope (FESEM, JEOL 7600).

### Reusability of immobilised enzyme

The reusability was assessed over 15 cycles of repeated usage. The original activity was assumed as the 100% control so that the percentage activity could be gauged after repeated use.

### Storage stability

The free and immobilised enzymes were stored for 60 days at 4 °C. The residual activity was assayed under the optimum conditions described above.

### Physicochemical characterisation

Temperatures from 35 to 85 °C and pHs from 4.0 to 8.5 were investigated to determine the optimal temperature and pH for the free and immobilised α-amylase. Thermal stability was investigated by assessing the relative activity of free and immobilised α-amylase after 30 minutes of incubation at temperatures from 35 to 85 °C before the addition of the substrate. The percentage relative activity versus temperatures was plotted. A Lineweaver-Burk plot with different concentrations of the substrate starch was used to gauge the *K*_m_ value.

### Influence of metal ions

The impact of metal ions on the activity of free and immobilized α- amylase was determined through incubation with 5 mM metal ion for 15 minutes before the addition of the starch to the mixture. The control (100%) was set as the activity of α- amylase in the absence of metal ion.

### Effect of metal chelators and inhibitors

The activity of free and immobilised α- amylase was determined under the influence of Ethylenediaminetetraacetic acid(EDTA), sodium oxalate, sodium citrate and metal chelators, 1,10 phenanthroline monohydrate, inhibitors 5,5′-dithiobis (2-nitrobenzoic acid) (DTNB), and phenylmethylsulfonyl fluoride(PMSF)at a concentration of 2 mM. The enzyme activity without the addition of these compounds was taken as 100% and the % relative activity in their presence was determined.

## Results and Discussion

**Figure 1:**
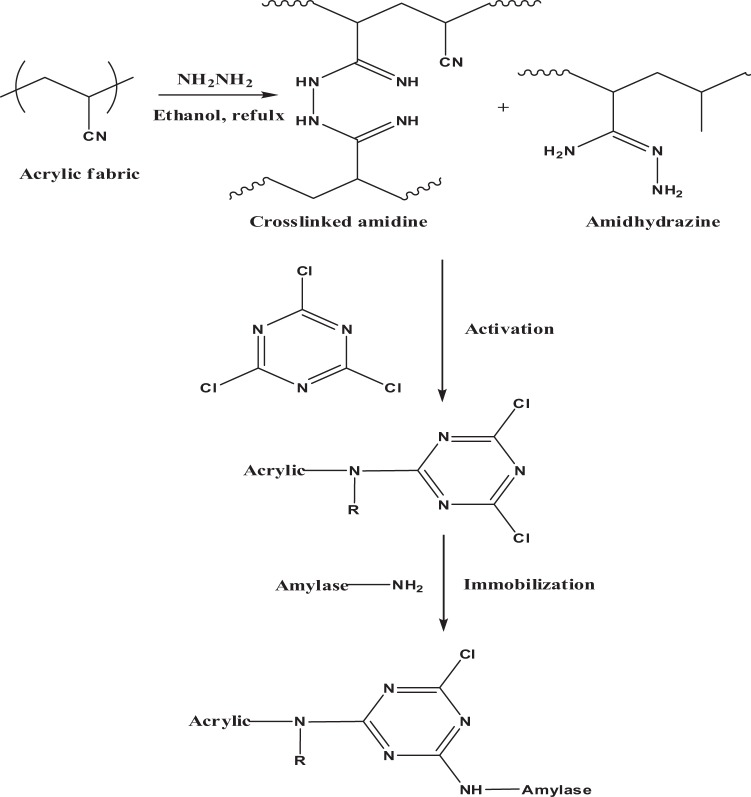
Hydrazine treatment, activation and immobilisation of α-amylase onto acrylic fabric.

The existence of surface functional groups and the bonding of α- amylase to the amidrazone acrylic fabric activated with cyanuric chloride were confirmed by ATR-FTIR spectroscopy and scanning electron microscopy (SEM). Figure 1 illustrates the method used for the amidrazone activation of the acrylic fabric for the immobilisation of α-amylase. The treated fabric was user-friendly and had numerous easily derivatised functional groups, good mechanical and chemical stability, and good capacity for enzyme immobilisation.

**Figure 2:**
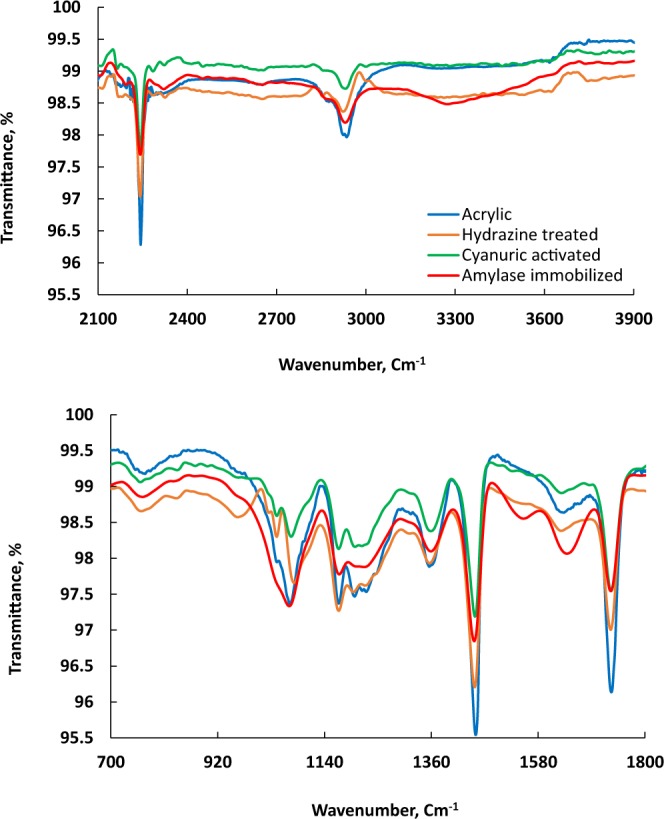
ATR-FTIR spectra of untreated, hydrazine-treated, cyanuric-activated and amylase-immobilised acrylic samples.

The ATR-FTIR spectra of the acrylic, hydrazine-treated, cyanuric-activated and amylase-immobilised samples are presented in Fig. 2. The first three samples exhibit similar absorption peaks with slight differences, whereas the spectrum of the amylase-immobilised sample is remarkably different. Characteristic broad bands owing to overlapped OH and NH groups present in the amylase enzyme are evident at 3279 cm^−1^ in the immobilised enzyme sample, while CH stretching vibration mode is observed around 2934 cm^−1^ in all of the samples^19–21^. Additionally, amide bands are also observed for the immobilised enzyme sample at 1646 cm^−1^ (CO stretch, amide I), 1553 cm^−1^ (NH bend, amide II) and at 1224 cm^−1^ (weak band, CN stretch, amide III). The success of the hydrazine treatment is clearly indicated by the decrease in the intensity of the nitrile absorption peak in the spectrum of the hydrazine-treated sample compared with the untreated acrylic fabric as the nitrile group is partially converted to amidrazone and/or cross-linked amidine (Fig. 1). Furthermore, a clear CN stretching vibration peak is observed at 969 cm^−1^ in the hydrazine-treated sample. The presence of carboxylate groups in the acrylic fabric is also indicated by the C=O peak at 1732 cm^−1^ and the C–O–C stretching vibration at 1071 cm^−1^. These peaks were either shifted or, their intensity changed upon hydrazination, cyanuric activation and α-amylase immobilisation. Cyanuric activation takes place via nucleophilic substitution reaction with NH and/or NH_2_ groups present in the treated samples (Fig. 1), and thus the broad peak centred at 3370 cm^−1^ would change upon reaction of the treated sample with cyanuric chloride, as shown in Fig. 2. In addition, the vibrations observed at 850 and 1530 cm^−1^ for the C–Cl bond and the triazinyl ring, respectively, could indicate the presence of a dichlorotriazinyl ring. Overall, the peak changes and the appearance of amylase peaks in the FTIR spectra indicate that the amylase immobilization was successful.

**Figure 3:**
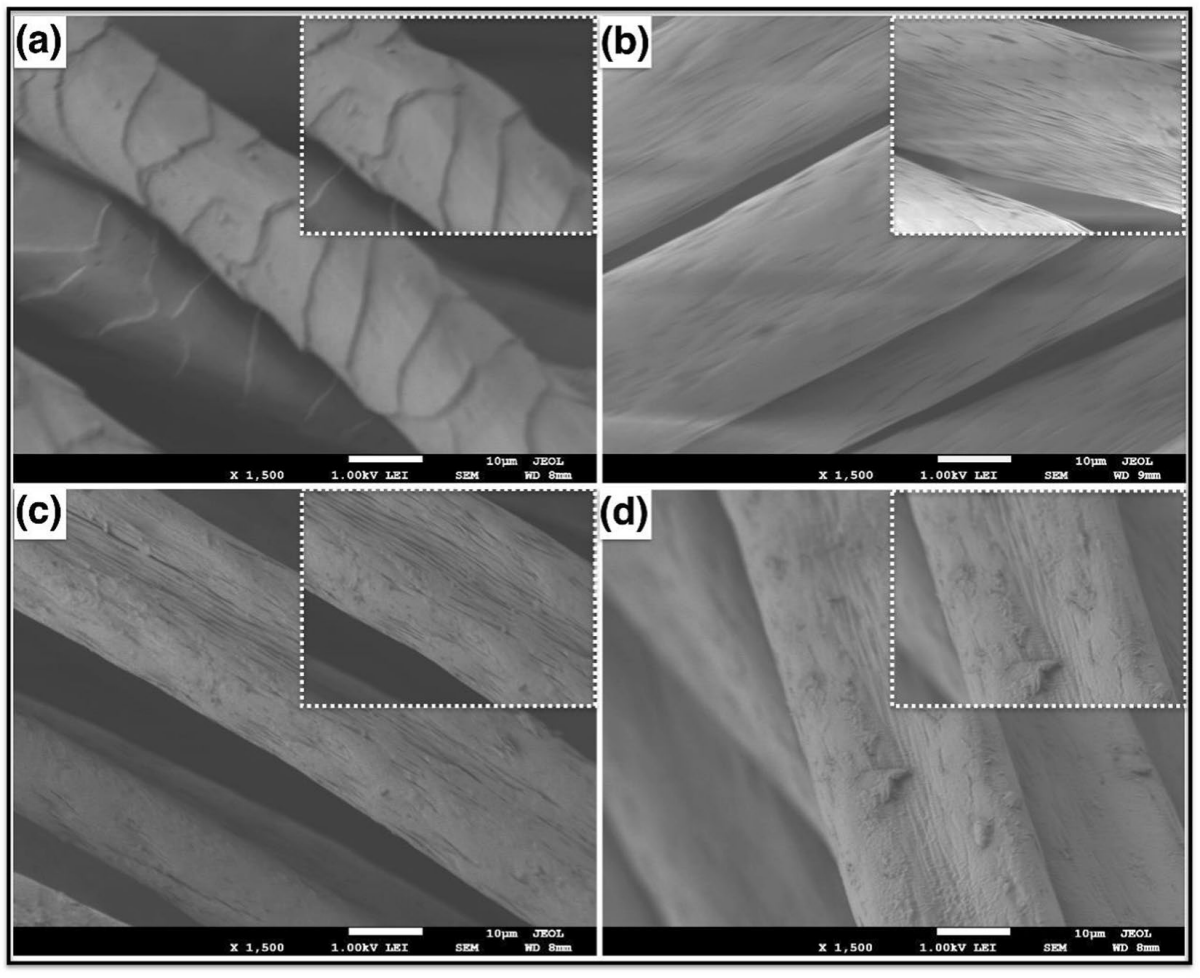
Low and high magnification FESEM images of (**a**) pristine acrylic fabric, (**b**) acrylic fabric treated with hydrazine, (**c**) hydrazine-acrylic fabric treated with cyanuric chloride, (**d**) immobilisation of α-amylase after chemical treatment.

The morphology of the surface of the untreated acrylic, hydrazine-treated, cyanuric-activated and amylase- immobilised samples was studied by SEM (Fig. 3). As can be seen in Fig. 3a, the untreated acrylic fabric exhibited a dermis-like structure on the surface. Significant differences were observed on the surface of the acrylic fabric after treatment with hydrazine hydrochloride, as shown in Fig. 3b. The surface structure of the hydrazine-treated acrylic appears to be more compact and exhibits a smooth surface. Moreover, after treatment with cyanuric chloride, the morphology of the acrylic fabric was still retained but minor changes could be observed, such as small cracks and defects, as shown in Fig. 3c. After immobilisation of the enzyme (Fig. 3d), it can be seen that the enzyme was dispersed on the surface of the acrylic fabric in different locations, indicating the successful immobilisation of the enzyme.

**Figure 4:**
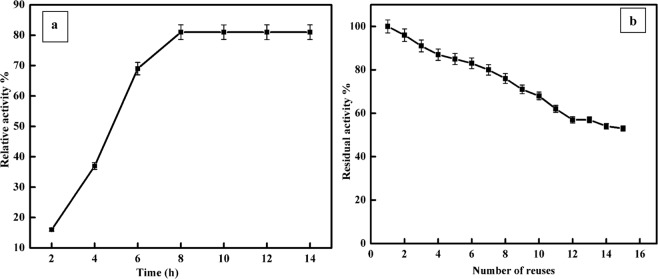
(**a**) Effect of immobilisation time on the relative activity of the immobilised α-amylase. (**b**) Reuse of immobilised α-amylase. Each point represents the mean of three experiments ± S.E.

α-Amylase immobilisation on amidrazone acrylic fabric activated with varying concentrations of cyanuric chloride (2, 4, 6%) at immobilisation pHsof4.0, 7.0 and 8.0 was evaluated. The maximum immobilisation efficiency of α-amylase was 81% for the sample activated with 4% cyanuric chloride at an immobilisation pH of7.0 (Table 1). The immobilisation efficiencies decreased in the order: 4% > 2% > 6% cyanuric chloride concentration. At a high concentration of cyanuric chloride, one would expect that excess enzyme attachment sites, might reduce the activity of the enzyme owing to changes to its stereochemical configuration. This result is in agreement with previous reports regarding the effects of cyanuric chloride on enzyme activity^22,23^. The effect of immobilisation time on the relative activity of the immobilised enzyme is presented in Fig. 4a. Clearly, the immobilised enzyme’s activity initially experienced a rapid increase with immobilisation time, reaching a plateau after 8 hours as a result of saturation. An important factor in industrial use is enzyme reusability. α- Amylase that has been immobilised on amidrazone acrylic fabric can be separated from the reaction mixture very easily making it possible to reuse the enzyme multiple times for starch hydrolysis. The activity of the immobilised α- amylase was examined over 15 reaction cycles to determine the reusability (Fig. 4b). As shown in the figure, 53% of the initial enzyme activity was retained after 15 reuses, which is superior to the 21.4% obtained by Ahmed^24^. These results indicate that the immobilised α-amylase has sufficient stability and reusability. The reduction of the enzyme activity upon repeated usage could be attributed primarily to two factors: the change in the stereochemical structure of the enzyme and leaching of the enzyme during the recycling process. Moreover, the frequent interaction of the substrate with the active site of the immobilised enzyme causes distortion of the active site, leading to loss of activity^25,26^.

**Table 1:** Effect of cyanuric chloride concentration and pH on α-amylase immobilisation efficiency. Each entry is the mean of three experiments ± S.E.

Cyanuric chloride (%)	Immobilization efficiency (%)		
	pH 8	pH 7	pH 4
2	48 ± 0.064	52 ± 0.105	31 ± 0.019
4	36 ± 0.032	81 ± 0.141	18 ± 0.042
6	27 ± 0.096	43 ± 0.084	11 ± 0.021

**Figure 5:**
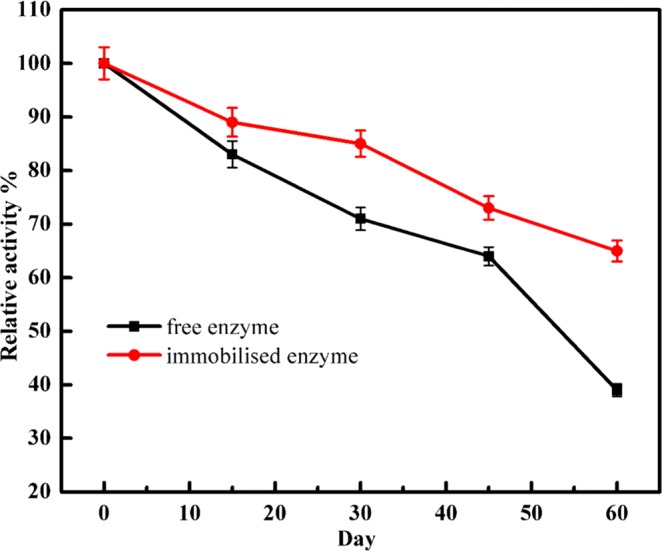
Storage stability of free and immobilised α-amylase. Each point represents the mean of three experiments ± S.E.

To evaluate the storage stability, the activities of the immobilised and free enzymes were evaluated after being stored at 4 °C for a period of 60 days. The catalytic activity was measured every 15 days. The results showed that the enzyme’s storage stability was enhanced by the immobilisation (Fig. 5) with the immobilised α-amylase retaining 65% of its original activity after 8 weeks, while the free enzyme retained 39% of its original activity. Thus, the immobilisation significantly prevented enzyme deactivation and enhanced the enzyme’s storage stability.

**Figure 6:**
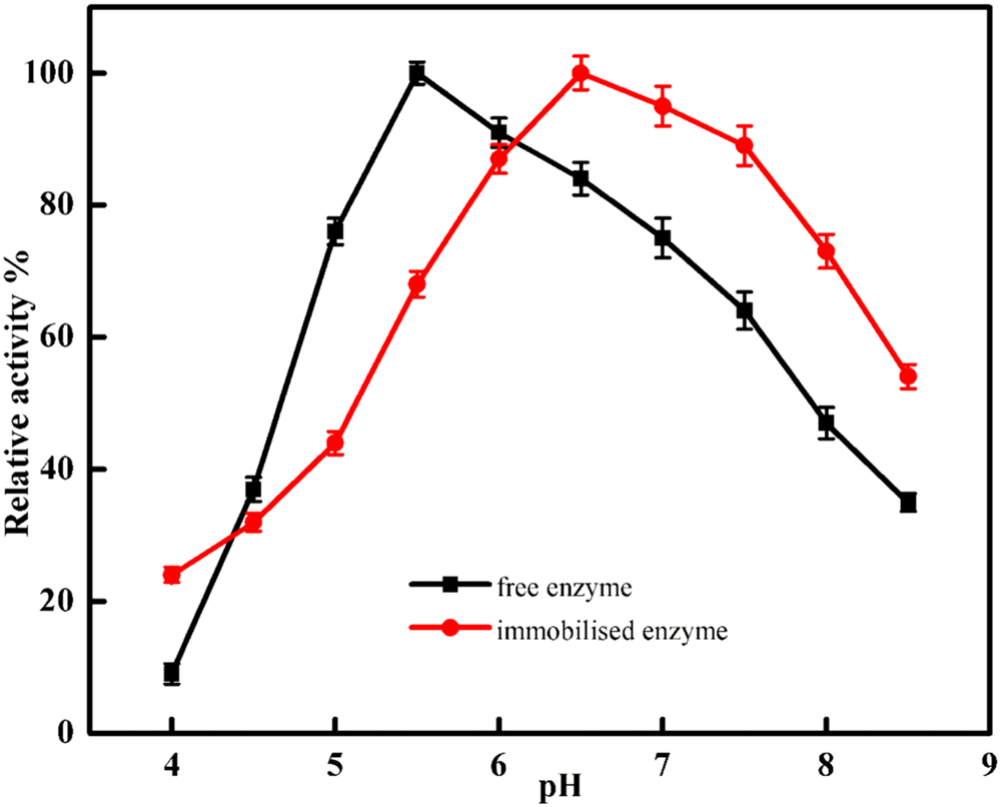
Optimum pH for free and immobilised α-amylase. Each point represents the mean of three experiments ± S.E.

**Figure 7:**
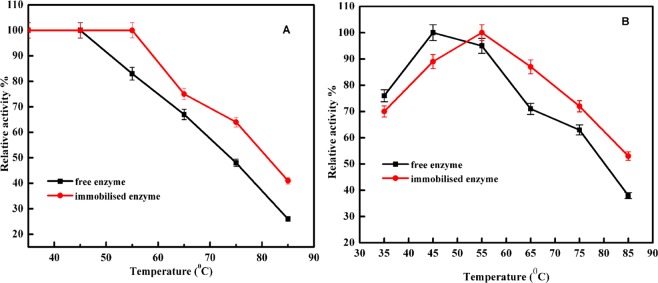
(**a**) Thermal stability and (**b**) optimum reaction temperature of free and immobilised α-amylase. Each point represents the mean of three experiments ± S.E.

**Figure 8:**
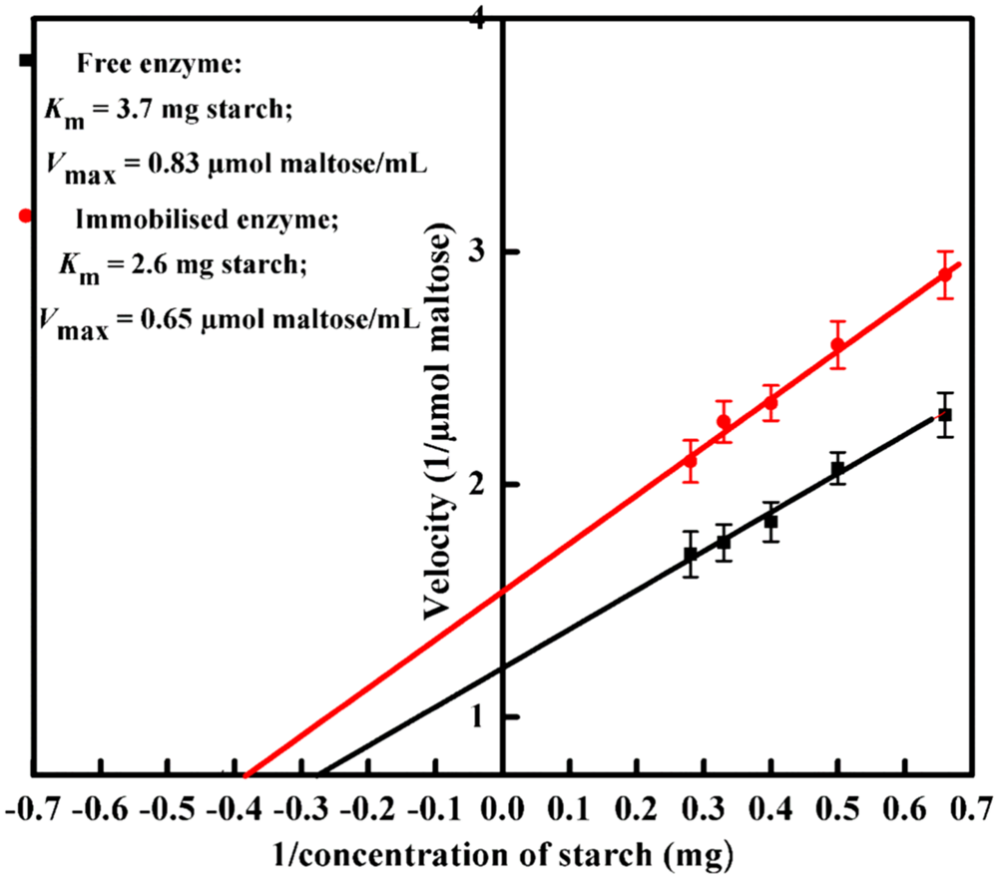
*K*_m_ offree and immobilised α-amylase.Each point represents the mean of three experiments ± S.E.

The immobilised and free enzymes exhibited their maximum activity at pH 6.5 and 5.5, respectively (Fig. 6), in agreement with the results reported from other studies^5,27^. The change of the optimal pH may be attributed to immobilized method, support structure and conformation change of enzyme after bounded. The immobilised α-amylase retained 95 to 54% of its activity at pH range 7.5 to 8.5. For industrial application of α-amylase, it is important that the immobilised enzyme has good thermal stability^28^. The results presented in Fig. 7a show that the immobilised α-amylase has improved thermal stability compared to that of the free enzyme^29,30^. Free α- amylase exhibited the highest activity at 45 °C, while immobilisation caused this temperature to rise to 55 °C. The results show that amidrazone acrylic fabric has a protective effect on α- amylase activity at higher temperatures. The development of many covalent bonds between the support and the enzyme reduces conformational flexibility, thermal vibrations, and enzyme mobility, while also preventing aggregation and unfolding of the enzyme protein^31^. The reduction of the relative activity of the unbound enzyme was reduced by thermal denaturation at higher temperatures, whereas the immobilised enzyme’s activity reduced more slowly above 55 °C. This is because the three-dimensional structure of the protein is protected from thermal denaturation by the restriction of the enzyme mobility upon immobilisation^32^. Upon varying the temperature in the range of 50–80 °C, the free enzyme was observed to be more temperature-sensitive than the immobilised one, indicating the usefulness of immobilisation (Fig. 7b). Similarly, Pereira *et al*.^5^ and Mohamed *et al*.^33^ reported the optimum temperature for immobilised α-amylase to be 50 °C. This increase in thermal stability and optimum working temperature for the immobilised enzyme might be attributed to changes in the stereochemical configuration of the enzyme^30,32^. The relation between the rate of the enzymatic reaction and the substrate concentration can be displayed using a Lineweaver–Burk plot (Fig. 8). The apparent *K*_m_ for the free and immobilised α-amylase was found to be 3.7 and 2.6 mg starch/mL, respectively. The decrease in *K*_m_ for the immobilised enzyme shows that the enzyme’s affinity for starch increased by 1.4-fold as a result of immobilisation, and thus the activity also increased. The α-amylase molecule might be extended over the supporting surface with an improved orientation, leading to more available active sites and a higher affinity for the substrate^34^. The apparent *V*_max_ values for the free and immobilised α-amylase were found to be 0.83 and 0.65 µmol maltose/mL, respectively. The fall in *V*_max_ seen after immobilisation is in line with the mass transfer restriction of the diffusion layer that surrounds the enzyme particles^31^, which limits the diffusion of the starch towards amidrazone acrylic fabric-immobilised α-amylase. It can also be explained through the consistent orientation of the enzyme molecules on the amidrazone acrylic fabric surface, which leads to a considerable fall in the affinity of the enzyme^35^. The observed changes to the catalytic properties could also result from modifications to the three-dimensional protein conformation caused by the enzyme binding to the support. Similar results were reported by Eslamipour and Hejazi^30^ and Homaei and Saberi^36^ for free and immobilised α-amylase.

Table 2 illustrates the influence of metal ions on the activity of free and immobilised α-amylase. As can be seen from this table, the immobilised α-amylase is highly stable in the presence of Ca^2+^, Ni^2+^ and Zn^2+^, retaining 120, 123 and 108% of its activity, respectively. The other metal ions tested had a partial inhibitory effect on immobilised α-amylase compared with the free enzyme. Several studies have reported that immobilisation protects α-amylase against heavy metal ion inhibition^27,33,37^. Immobilised α-amylase presented noteworthy stability in the presence of the tested inhibitors (Table 3). However, the free enzyme was not stable under the same conditions.

**Table 2:** Effect of metal ions (5 mM) on the activity of free and immobilised α-amylase. Each entry is the mean of three experiments ± S.E.

Metals ions	Free α-amylase	Immobilised α-amylase
control	100 ± 1.46	100 ± 1.23
Ca^2+^	86 ± 0.79	120 ± 1.15
Ni^2+^	102 ± 1.18	123 ± 1.64
Cd^2+^	54 ± 0.42	81 ± 0.87
Zn^2+^	98 ± 0.69	108 ± 0.89
Co^2+^	63 ± 0.58	76 ± 0.96
Pb^2+^	21 ± 0.35	49 ± 0.37
Hg^2+^	15 ± 0.73	37 ± 0.51

**Table 3:** Effect of inhibitors (2 mM) on the activities of the free and immobilised α-amylase. Each entry is the mean of three experiments ± S.E.

Inhibitor	Free α-amylase	Immobilized α-amylase
EDTA	23 ± 0.006	63 ± 0.046
Sodium Oxalate	53 ± 0.018	81 ± 0.011
Sodium Citrate	41 ± 0.027	68 ± 0.038
DTNB	48 ± 0.064	79 ± 0.017
1,10 phenatroline	54 ± 0.075	86 ± 0.028
PMSF	37 ± 0.081	65 ± 0.061

## Conclusion

This report describes the immobilisation of α-amylase by cross-linking and covalent bonding on acrylic fabric with amidrazone that has been activated using cyanuric chloride as a new organic support. The immobilisation of the enzyme on the carrier improved the thermal stability, pH levels and affinity for starch. Furthermore, the enzyme’s storage stability and reusability were also improved by immobilisation. The immobilised enzyme can be separated with great ease from the reaction mixture, after which it is can be used again in a fresh enzymatic reaction. Confirmation that the α-amylase was attached to the treated acrylic fabric was obtained by FTIR and SEM. In conclusion, amidrazone acrylic fabric has been demonstrated to be an excellent carrier for industrial enzymes and the authors recommend its use for enzyme immobilisation. It should prove useful for industrial applications involving biopharmaceuticals, biocatalysts and biotechnology.

## References

[1] Jemli S, Ayadi-Zouari D, Hlima HB, Bejar S (2016). Biocatalysts: application and engineering for industrial purposes. Crit. Rev. Biotechnol..

[2] Kirk O, Borchert TV, Fuglsang CC (2002). Industrial enzyme applications. Curr. Opin. Biotechnol..

[3] Reddy NS, Nimmagadda A, Rao KS (2003). An overview of the microbial α-amylase family. Afr. J. Biotechnol..

[4] Ahmad R, Mohsin M, Ahmad T, Sardar M (2015). Alpha amylase assisted synthesis of TiO2 nanoparticles: structural characterization and application as antibacterial agents. J. Hazar. Mater..

[5] Pereira SE, Fernandes KF, Ulhoa CJ (2017). Immobilization of Cryptococcus flavus α‐amylase on glass tubes and its application in starch hydrolysis. Starch‐Stärke.

[6] Rodriguez J, Soria F, Geronazzo H, Destefanis H (2016). Modification and characterization of natural aluminosilicates, expanded perlite, and its application to immobilise α–amylase from A. *oryzae*. J. Mol. Catal. B: Enzym..

[7] Luo X, Zhang L (2010). Immobilization of penicillin G acylase in epoxy-activated magnetic cellulose microspheres for improvement of biocatalytic stability and activities. Biomacromolecules.

[8] DiCosimo R, McAuliffe J, Poulose AJ, Bohlmann G (2013). Industrial use of immobilized enzymes. Chem. Soc. Rev..

[9] Iyer PV, Ananthanarayan L (2008). Enzyme stability and stabilization—aqueous and non-aqueous environment. Process Biochem..

[10] Madhu A, Chakraborty JN (2017). Developments in application of enzymes for textile processing. J. Clean. Prod..

[11] Pandey G, Munguambe DM, Tharmavaram M, Rawtani D, Agrawal YK (2017). Halloysite nanotubes-An efficient ‘nano-support’for the immobilization of α-amylase. Appl. Clay Sci..

[12] Asgher M, Shahid M, Kamal S, Iqbal HMN (2014). Recent trends and valorization of immobilization strategies and ligninolytic enzymes by industrial biotechnology. J. Mol. Catal. B: Enzym..

[13] Kallenberg AI, van Rantwijk F, Sheldon RA (2005). Immobilization of penicillin G acylase: the key to optimum performance. Adv. Synth. Catal..

[14] Audrieth, L. F. & Ogg, B. A. *The Chemistry of Hydrazine* 1244 (John Wiley, 1951)

[15] Bottomley F (1970). The reactions of hydrazine with transition-metal complexes. Quarterly Reviews. Chem. Soc. Rev.

[16] Schmidt, E. W. *Hydrazine and its Derivatives*(Wiley-Interscience, 2001).

[17] Heaton BT, Jacob C, Page P (1996). Transition metal complexes containing hydrazine and substituted hydrazines. Coord. Chem. Rev..

[18] Miller GL (1959). Use of dinitrosalicylic acid reagent for determination of reducing sugar. Anal. Chem..

[19] Mohamed SA, Al-Ghamdi SS, El-Shishtawy RM (2016). Immobilization of horseradish peroxidase on amidoximated acrylic polymer activated by cyanuric chloride. Int. J. bio. Macromol..

[20] Mohamed SA, Darwish AA, El-Shishtawy RM (2013). Immobilization of horseradish peroxidase on activated wool. Process Biochem..

[21] Mohamed SA, Al-Malki AL, Kumosani TA, El-Shishtawy RM (2013). Horseradish peroxidase and chitosan: activation, immobilization and comparative results. Int. J. bio. Macromol..

[22] Cao L (2005). Immobilised enzymes: science or art?. Curr. Opin. Chem. Bio..

[23] Brady D, Jordaan J (2009). Advances in enzyme immobilisation. Biotechnol. Let..

[24] Ahmed SA, Mostafa FA, Ouis MA (2018). Enhancement stability and catalytic activity of immobilized α-amylase using bioactive phospho-silicate glass as a novel inorganic support. Int. J. bio. Macromol..

[25] Qiu H (2009). Immobilization of laccase on nanoporous gold: comparative studies on the immobilization strategies and the particle size effects. J. Phys. Chem. C.

[26] Defaei M, Taheri-Kafrani A, Miroliaei M, Yaghmaei P (2018). Improvement of stability and reusability of α-amylase immobilized on naringin functionalized magnetic nanoparticles: A robust nanobiocatalyst. Int. J. bio. Macromol..

[27] Mohamed SA, Khan JA, Al-Bar OA, El-Shishtawy RM (2014). Immobilization of trichoderma harzianum α-Amylase on treated wool: Optimization and characterization. Molecules.

[28] Veesar IA, Solangi IB, Memon S (2015). Immobilization of α-amylase onto a calix [4] arene derivative: Evaluation of its enzymatic activity. Bioorg. Chem..

[29] He T, Tian YL, Qi L, Zhang J, Zhang ZQ (2014). Improved performance of α-amylase immobilized on poly (glycidyl methacrylate-co-ethylenedimethacrylate) beads. Int. J. Biol. Macromol..

[30] Eslamipour F, Hejazi P (2016). Evaluating effective factors on the activity and loading of immobilized α-amylase onto magnetic nanoparticles using a response surface-desirability approach. RSC Adv..

[31] Gashtasbi F, Ahmadian G, Noghabi KA (2014). New insights into the effectiveness of alpha-amylase enzyme presentation on the Bacillus subtilis spore surface by adsorption and covalent immobilization. Enzyme Microb. Technol..

[32] Antony N, Balachandran S, Mohanan PV (2016). Immobilization of diastase α-amylase on nano zinc oxide. Food chem..

[33] Mohamed, S. A. *et al*. Immobilization of Trichoderma harzianum α-amylase on PPyAgNp/Fe3O4-nanocomposite: chemical and physical properties. *Artif. Cells, Nanomed. Biotechnol*., 1–6 (2018). 10.1080/21691401.2018.1453828 29578361

[34] Sohrabi N, Rasouli N, Torkzadeh M (2014). Enhanced stability and catalytic activity of immobilized α-amylase on modified Fe3O4 nanoparticles. Chem. Eng. J..

[35] Akkaya B, Yenidunya AF, Akkaya R (2012). Production and immobilization of a novel thermoalkalophilic extracellular amylase from bacilli isolate. Int. J. Biol. Macromol..

[36] Homaei A, Saberi D (2015). Immobilization of α-amylase on gold nanorods: An ideal system for starch processing. Process Biochem..

[37] Pascoal AM, Mitidieri S, Fernandes KF (2011). Immobilisation of α-amylase from Aspergillus niger onto polyaniline. FBP.

